# Renal Metastasis of Osteosarcoma with IVC Thrombus

**DOI:** 10.1155/2021/8882593

**Published:** 2021-03-20

**Authors:** Prem Raj Sigdel, Diwas Gnyawali, Purushottam Parajuli, Sampanna Chudal, Durga Pandit, Bipin Guragain, Manish Man Pradhan, Sujeet Poudyal, Suman Chapagain, Bhoj Raj Luitel, Pawan Raj Chalise, Uttam Kumar Sharma

**Affiliations:** Department of Urology and Kidney Transplant Surgery, Tribhuvan University Teaching Hospital, Institute of Medicine, Maharajgunj, Kathmandu 44600, Nepal

## Abstract

Renal metastasis from osteosarcoma is a rare entity, and tumour thrombus is even rarer. To date, only 15 cases of osteosarcoma with tumour thrombus have been reported in the literature. We present a case of an 18-year-old female diagnosed as having right distal femur osteosarcoma, later presenting with renal osteosarcoma with IVC thrombus.

## 1. Introduction

Renal metastasis from osteosarcoma is a rare entity, and tumour thrombus is even rarer. We present a case of an 18-year-old female who was diagnosed with right distal femur osteosarcoma who developed metastasis in the bilateral lungs one year later and renal metastasis after 1.5 years. Osteosarcoma with metastasis to the kidney with IVC thrombus is an aggressive tumour and has a poor prognosis. A multidisciplinary approach is required for the appropriate management.

## 2. Case Presentation

An eighteen-year-old female was diagnosed as having a case of right distal femur osteosarcoma 1.5 years back. The patient underwent resection of the femur with the placement of a prosthesis. Biopsy reports of the femur showed chondroblastic osteosarcoma. She received four cycles of cisplatin-based chemotherapy postoperatively. On routine CT chest after one year, nodular lesions were seen in bilateral lungs. Biopsy of these lung lesions showed metastatic osteosarcoma. She underwent right lower lobectomy and left upper lobe metastasectomy.

**Figure 1:**
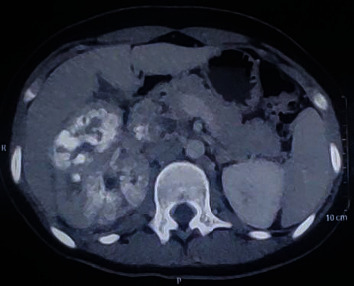
Axial CT scan showing right renal calcifications with involvement of IVC.

**Figure 2:**
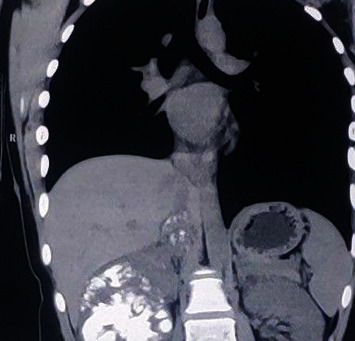
Coronal CT scan showing the involvement of IVC with the tumour thrombus.

Six months after the lung lesions, she presented to us with complaints of vague abdominal pain. She had no haematuria or abdominal mass. She had no respiratory, urinary, or other systemic symptoms. CECT abdomen and pelvis showed right enlarged kidney with calcifications with calcified thrombus in IVC (Figures 1 and 2). CT head was done, which showed no brain metastasis.

**Figure 3:**
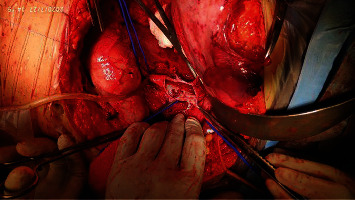
Cavotomy and removal of tumor thrombus.

**Figure 4:**
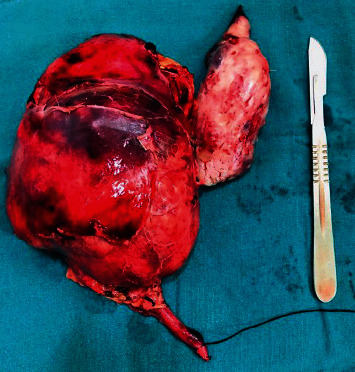
Specimen of the right kidney and IVC thrombus.

A multidisciplinary discussion was done with provisional diagnosis of metastatic renal sarcoma with level III thrombus. She was planned for right radical nephrectomy and IVC thrombectomy. Intraoperatively, kidney mobilization and hilar dissection were done. After liver mobilization, sequential IVC clamping was done in order or infrarenal IVC, left renal vein, Pringle manoeuvre, and suprahepatic IVC. Venacavotomy and thrombus retrieval were done (Figure 3). The specimen was retrieved (Figure 4). Closure of cavotomy with sequential declamping was done. Postoperative events were uneventful.

**Figure 5:**
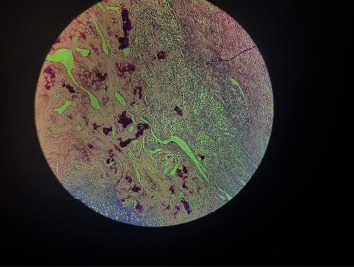
Histopathology showing renal osteosarcoma.

Histopathology reports showed metastatic osteosarcoma of the kidney (Figure 5). Patients were lost to follow-up in our centre. We later knew that she developed metastatic deposits in the pelvic bones one month after surgery, but the MRI images are not available.

## 3. Discussion

Osteosarcoma is the most frequent malignant tumour of bones, with a peak incidence in the second decade of life [1]. The metaphyseal part of long bones is most frequently involved. The most common site of metastasis of osteosarcoma is the lung. Metastasis to soft tissues and solid organs is rare. Extrapulmonary sites are more common in treated cases because of change in the natural history of the disease by more prolonged survival of these patients and the use of multiple chemotherapy agents [2]. To date, only 15 cases of osteosarcoma with tumour thrombus have been reported in the literature [3]. The diagnosis of IVC thrombus is usually incidental. The reported incidence of renal metastasis of extrarenal neoplasms varies from 2 to 20% [4]. Metastatic osteosarcoma is usually diagnosed by imaging because of concern of dissemination of disease on needle biopsy [5]. Metastatic lesions usually do not have enough calcifications that can be seen in simple radiograph as compared to primary renal osteosarcoma [6]. Renal metastasis of osteosarcoma is generally detected after death as part of widespread disease; 10–12% of patient autopsies showed renal involvement [4]. Surgical resection of the intravascular/intracardiac tumour thrombus is the treatment of choice. Patients with metastatic tumour thrombus generally do not survive for more than 12 or 13 months [3].

## 4. Conclusion

Osteosarcoma with renal metastasis with tumour thrombus is a rare entity. Successful management of such cases relies on a careful multidisciplinary approach. Whether surgery increases the survival of these patients is still unknown and requires further investigations.

## Data Availability

The data used to support the findings of this study are included in the article.
